# Microbial and enzymatic activity of soil contaminated with azoxystrobin

**DOI:** 10.1007/s10661-015-4827-5

**Published:** 2015-09-07

**Authors:** Małgorzata Baćmaga, Jan Kucharski, Jadwiga Wyszkowska

**Affiliations:** University of Warmia and Mazury in Olsztyn, Plac Łódzki 3, 10-727 Olsztyn, Poland

**Keywords:** Azoxystrobin, Microorganisms, Biodiversity, Enzymes, Resistance, Identification of microorganisms

## Abstract

The use of fungicides in crop protection still effectively eliminates fungal pathogens of plants. However, fungicides may dissipate to various elements of the environment and cause irreversible changes. Considering this problem, the aim of the presented study was to evaluate changes in soil biological activity in response to contamination with azoxystrobin. The study was carried out in the laboratory on samples of sandy loam with a pH of 7.0 in 1 Mol KCl dm^−3^. Soil samples were treated with azoxystrobin in one of four doses: 0.075 (dose recommended by the manufacturer), 2.250, 11.25 and 22.50 mg kg^−1^ soil DM (dry matter of soil). The control soil sample did not contain fungicide. Bacteria were identified based on 16S rRNA gene sequencing, and fungi were identified by internal transcribed spacer (ITS) region sequencing. The study revealed that increased doses of azoxystrobin inhibited the growth of organotrophic bacteria, actinomycetes and fungi. The fungicide also caused changes in microbial biodiversity. The lowest values of the colony development (CD) index were recorded for fungi and the ecophysiological (EP) index for organotrophic bacteria. Azoxystrobin had an inhibitory effect on the activity of dehydrogenases, catalase, urease, acid phosphatase and alkaline phosphatase. Dehydrogenases were found to be most resistant to the effects of the fungicide, while alkaline phosphatase in the soil recovered the balance in the shortest time. Four species of bacteria from the genus *Bacillus* and two species of fungi from the genus *Aphanoascus* were isolated from the soil contaminated with the highest dose of azoxystrobin (22.50 mg kg^−1^).

## Introduction

Pesticides are chemicals which play a major role in maintaining adequate quality of agricultural products by controlling plant pathogens. In addition, they are used in human and animal hygiene, in the protection of feed, food, natural raw materials and products made of them (Chatterjee et al. 2013). Primarily, the use of pesticides is aimed at controlling target organisms. Despite this fact, it is not possible to predict the environmental fate of pesticides. The widespread use, toxicity, mobility and persistence of pesticides may lead to their dissipation to all elements of the natural environment. Thus, the excessive use of pesticides is still a major problem affecting the quality of the natural environment, which is why more and more studies are being conducted to determine their effects on living organisms (Seiber and Kleinschmidt 2011; Wyszkowska and Kucharski 2004). The highest quantities of pesticides are accumulated in soil, which may cause changes in the terrestrial environment, often manifested by decreasing soil fertility. Studies on the presence of pesticides in soil and their impact on soil organisms are necessary because soil is most affected by contamination. Microbial activity of soil is used to assess the potential changes caused by these chemicals. Microbial response to contaminants dissipating to soil is prompt and thus can provide necessary information on environmental changes (Zhang et al. 2006). Due to the fact that plant protection products are potentially harmful to non-target organisms, there has been considerable interest in determining their impact on soil microorganisms and processes (Bending et al. 2007).

Azoxystrobin (methyl(*E*)-2-{2-[6-(2-cyanophenoxy)pyrimidin-4-yloxy]phenyl}-3-methoxyacrylate), a strobilurin-derived fungicide, is one of the most popular chemicals used for the control of fungal plant pathogens. It has a broad spectrum of systemic activity against pathogens by inhibiting mitochondrial respiration in the process of binding with cytochrome b complexes. The binding process blocks electron transport from cytochrome b to c, thus inhibiting the generation of energy through the oxidative phosphorylation necessary for cell growth, and finally causes the death of the pathogen (Bartlett et al. 2002). In the natural environment, azoxystrobin is degraded to azoxystrobin acid, which is much more water-soluble and prone to leaching in soils than its parent compound (Ghosh and Singh 2009). Rodrigues et al. (2013) reported that the half-life of azoxystrobin can range from 14 days to 6 months, depending on the microbiological and biochemical soil parameters. Microbial degradation of azoxystrobin is associated with the hydrolysis of the carboxylic ester bond in the parent compound (Katagi 2006). Therefore, microorganisms and enzymes have a significant role in the degradation of this active substance (Clinton et al. 2011).

The literature (Guo et al. 2015) provides limited information on the impact of azoxystrobin on microbial and biochemical activity in different types of soil. Considering this fact, the aim of our study was to determine the effect of azoxystrobin on the soil ecosystem by determining microbial counts, their biodiversity, enzymatic activity of soil, soil resistance and the soil resilience index. In the study, new microbial strains resistant to azoxystrobin were isolated.

## Materials and methods

### Soil

A laboratory experiment was carried out on samples of soil with the granulometric composition of sandy loam with a pH 7.0. The soils were classified as Eutric Cambisols based on the World Reference Base of Soil Resources (2014). The tested soils are characterized in Table 1. Soil samples were collected from the humus level at a depth of 0 to 20 cm, in Tomaszkowo near Olsztyn in north-eastern Poland. The physicochemical properties of the soil was determined by the following methods: grain-size composition of the soil with laser method using a Mastersizer 2000 (PB 33 ed.303.12.2012, 2012), soil pH by potentiometry in aqueous KCl solution of the concentration of 1 Mol KCl dm^−3^, hydrolytic acidity and total exchangeable bases by the Kappen’s method (Carter 1993), content of organic carbon—with Tiurin’s method (Nelson and Sommers 1996), content of total nitrogen according to the method by Kjeldahl (PB 29 ed.303.12.2012, 2012), as well as the content potassium, sodium, calcium and magnesium cations by flame photometry (BS EN ISO 11260, 2011).

**Table 1:** General characteristics of experimental soil

Parameter	Sandy loam
Sand (2000–50 μm) %	69.41
Silt (50–2 μm) %	27.71
Clay (<2 μm) %	2.88
pH_KCl_	7.00
HAC (mMol^(+)^ kg^−1^)	6.40
TEB (mMol^(+)^ kg^−1^)	165.9
C_org_ (g kg^−1^)	14.30
N_total_ (g kg^−1^)	0.98
Exchangeable cations (mg kg^−1^)	
K^+^	180.0
Ca^2+^	2571.4
Na^+^	20.0
Mg^2+^	59.5

*HAC* hydrolytic acidity, *TEB* total exchangeable bases, *C*_*org*_ organic carbon content, *N*_*total*_ total nitrogen content

### Fungicide

Azoxystrobin, which is the active ingredient to fungicide Amistar 250 SC, was tested in amount of 250 g dm^-3^. Azoxystrobin belongs to the group of strobilurins, which is designed against fungal diseases in agricultural crops and vegetables. This formulation made by Syngenta in an amount of from 0.8 to 1.0 dm^3^ ha^−1^. The predicted environmental concentrations (PEC) of azoxystrobin in soil on days 30, 60 and 90 are described in Table 2.

**Table 2:** Predicted environmental concentrations (PEC) azoxystrobin in soil, mg kg^−1^

Dose of azoxystrobin (mg kg^−1^)	Soil incubation time, days		
	30	60	90
0.075	0.0049	0.0038	0.0029
2.250	0.1465	0.1146	0.0896
11.50	0.7330	0.5731	0.4481
22.50	1.4660	1.1462	0.8961

### Experimental design

A laboratory experiment was performed in three replications, in 150 cm^3^ glass beakers each containing 100 g of air-dry soil. Before the experiment, soil was passed through a sieve with 2 mm mesh size. Each sample was treated with a single azoxystrobin as water emulsion in the following doses: 0.075 (dose recommended by the manufacturer), 2.250, 11.250, and 22.500 mg kg^−1^ soil DM. The control treatment comprised soil without fungicide. The soil was then combined with fungicide and brought to 50 % capillary capacity with the use of deionized water. Beakers were covered with perforated foil and incubated at 25 °C for 30, 60 and 90 days.

### Soil microorganisms

The counts organotrophic bacteria were determined in the Bunt and Rovira medium with the addition of soil extract (Alexander 1973), actinomycetes—in the Küster and Williams medium with the addition of antibiotics nystatin and actidione (Parkinson et al. 1971), and fungi grown on the Martin’s medium (1950) with added rose bengal and aureomycin. Plates were kept at 28 °C throughout the incubation period. Counts of colony forming units (cfu) were determined using a colony counter.

The azoxystrobin effect on the structure and biodiversity of organotrophic bacteria, actinomycetes and fungi was determined in soil samples. Diluted soil suspensions were incubated in Petri dishes, at 28 °C, in nine replications. Colonies were counted over a period of successive 10 days. Then calculated the colony development index CD = [N_1_/1 + N_2_/2 + N_3_/3….. N_10_/10] · 100, were N_1_, N_2_, N_3_,…. N_10_ counts of colonies that emerged on day 1, 2, 3, …, 10 (Sarathchandra et al. 1997), and the ecophysiological index EP = −∑(pi · log pi), were as follows: pi, counts of colonies that emerged on a given day divided by total counts of colonies (De Leij et al. 1993).

### Soil enzymes

Dehydrogenases (EC 1.1) activity, according to the method described by Öhlinger (1996), catalase (EC 1.11.1.6) and urease (EC 3.5.1.5) activity, according to the method by Alef and Nanipieri (1998) and acid phosphatase (EC 3.1.3.2) and alkaline phosphatase (EC 3.1.3.1), according to the method described by Alef et al. (1998), were determined in soil samples in nine replicates. Except for catalase, soil enzymatic activity was measured on a Perkin-Elmer Lambda 25 spectrophotometer (MA, USA) at *λ* = 485 nm (dehydrogenases) and *λ* = 410 nm (urease, acid phosphatase and alkaline phosphatase). The activity of catalase was determined based on the decomposition of hydrogen peroxide by potassium permanganate. The following substrates were used to determine enzymatic activity: 2,3,5-triphenyltetrazolium chloride for dehydrogenases (P.P.H Stanlab Sp.J, Poland), H_2_O_2_ for catalase H_2_O_2_ (P.P.H Stanlab Sp.J, Poland), urea for urease (Eurochem BGD Sp. z.o.o, Poland) and 4-nitrophenyl phosphate disodium for acid phosphatase and alkaline phosphatase (ApliChem GmbH, Germany). Enzymatic activity was expressed in the following units per kg DM h^−1^: μMol TPF for dehydrogenases, Mol O_2_ for catalase, mMol N-NH_4_ for urease, and mMol PNP for acid phosphatase and alkaline phosphatase.

Formulas proposed by Orwin and Wardle (2004) were used to determine the soil resistance index (RS) on days 30, 60 and 90, and the soil resilience (RL) index, indicating recovery of balance, on day 90 after contamination with azoxystrobin:

$$ RS\left({\mathrm{t}}_0\right)=\kern0.5em 1\kern0.5em \hbox{--} \frac{2\left|{\mathrm{D}}_0\right|}{\left({\mathrm{C}}_0+\left|{\mathrm{D}}_0\right|\right)} $$

where: C_0_ is the parameter value under natural conditions over time t_0_; P_0_ is the parameter value for soil disturbed over time t_0_, D_0_ = C_0_ - P_0_.

$$ RL\ \mathrm{at}\ {\mathrm{t}}_{\mathrm{x}}=\frac{2\left|{\mathrm{D}}_0\right|}{\left(\left|{\mathrm{D}}_0\right|+\left|\mathrm{D}\times \right|\right)}-1 $$

where: D_0_ = C_0_−P_0_, D_x_ = C_x_−P_x_, C_0_ is the parameter value under natural conditions over time t_0_, P_0_ is the parameter value for soil disturbed over time t_0_, C_x_ is the parameter value under natural conditions over time t_x_, P_x_ is the parameter value for soil disturbed over time t_x_.

### Isolation of DNA from bacteria and fungi and their identification

On day 90 of the experiment, bacteria and fungi were isolated from the soil free from fungicide, and samples were treated with either a 0.075 mg kg^−1^ (dose recommended by the manufacturer) or 22.50 mg kg^−1^ dose of azoxystrobin. Bacteria were isolated using PCA culture media (distilled water, 1 dm^3^; casein enzymatic hydrolysate, 5 g; yeast extract, 2.5 g; glucose, 1 g; agar, 15 g), and fungi were cultured on Sabouraud medium with chloramphenicol (distilled water, 1 dm^3^; yeast extract, 2 g; peptone, 3 g; peptone SP, 3 g; peptone K, 3 g; glucose, 19 g; dipotassium hydrogen phosphate, 0.5 g; potassium dihydrogen phosphate, 0.5 g; chloramphenicol, 0.5 g; agar, 15 g). Culture media containing an appropriate dilution of soil material (10^−5^ for bacteria and 10^−3^ for fungi) were incubated under aerobic conditions at 37 °C for 24–48 h. Characteristic colonies formed by the isolated microorganisms were passaged 10 times to obtain a pure culture. Genomic DNA was isolated using a Bead-Beat Micro Gravity kit (A&A Biotechnology, Poland), in line with the manufacturer’s instructions. Next, the isolated genomic DNA was separated electrophoretically in 1 % agarose gel (5 mm^3^ samples in gel). A PCR assay was carried out on a 50-mm^3^ sample, using a reaction mixture containing mm^3^ (∼50 ng) of genomic DNA from a sample, 25 mm^3^ 2 × PCR Master Mix Plus High GC (A&A Biotechnology), 0.2 mm^3^ of each primer, concentration 100 μM, and 19.6 mm^3^ sterile water. Conditions of the PCR assay on isolated DNA templates are presented in Table 3. The 16S rRNA gene sequence from bacterial DNA was amplified using a pair of primers: B-all For: GAG TTT GAT CCT GGC TCA G and B-all Rev.: ACG GCT ACC TTA CGA CTT, and the ITS region sequence from fungi was amplified using ITS1: TTC GTA GGT GAA CCT GCG G and ITS4: TCC TCC GCT TAT TGA TAT GC. PCR products were separated in 2 % agarose gel (2 mm^3^ samples in gel). DNA fragments obtained by amplification were purified using a Clean-Up AXE kit (A&A Biotechnology, Poland). PCR products were suspended in 10 mM Tris-HCl buffer, pH 8.0, diluted to a concentration of 100 ng mm^−3^ and sent to Macrogen (Netherlands) for sequencing. Identified sequences of 16S rRNA gene and ITS region were compared with sequences available in the GenBank database (National Center of Biotechnology Information) using Basic Local Alignment Search Tool (BLAST) analysis.

**Table 3:** Conditions of the PCR assay on isolated DNA templates

Reaction steps	Temperature		Time		Number of cycles	
	Bacteria	Fungi	Bacteria	Fungi	Bacteria	Fungi
Initial denaturation	94 °C	94 °C	2 min	2 min	1	1
Denaturation	94 °C	94 °C	30	30	30	30
Primer connection	58 °C	58 °C	45 s	45 s		
Elongation	72 °C	72 °C	1:30 min	1 min		
Final elongation	72 °C	72 °C	5 min	5 min	1	1

### Statistical analysis

Results were analysed statistically using the Statistica 10.0 package (Statsoft Inc. 2011). Homogeneous groups were identified using Tukey’s range test at a significance level of *p* = 0.01 by the analysis of variance (ANOVA). The soil microbial counts were estimated using Ward’s cluster analysis (CA). The phylogenetic tree of identified microbial strains was prepared in the MEGA 6.0 application by the “neighbour-joining” method. Resistance of soil (RS) to contamination with azoxystrobin was explained by principal component analysis (PCA). In addition, Pearson’s linear coefficients of correlation between the dose of azoxystrobin and the analysed parameters were calculated. The percentage of observed variance in microbial counts and enzymatic activity were calculated by two-way ANOVA using the *η*^2^ coefficient.

## Results and discussion

### Soil microorganisms

**Fig. 1:**
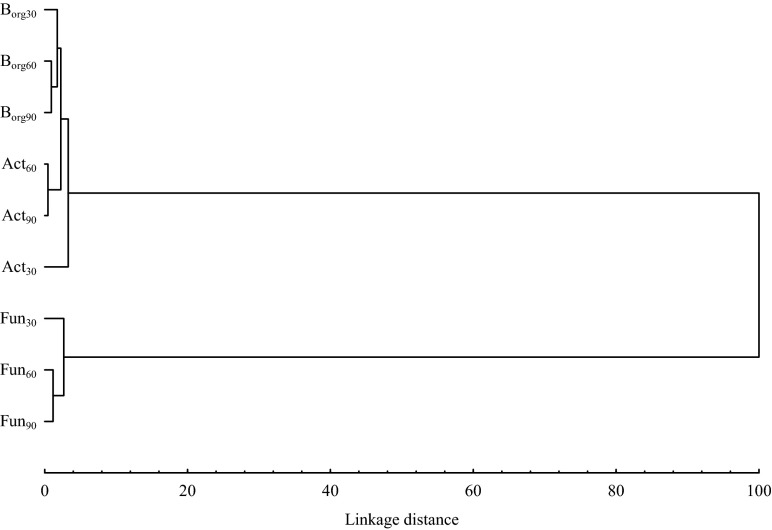
Similar response of microorganisms in soil contaminated with azoxystrobin. Explanation: *B*_*org*_ organotrophic bacteria; *Act* actinomycetes; *Fun* fungi; *30, 60 and 90* microorganisms counts at 30, 60 and 90 days of incubation soil

Microorganisms play a key role in soil biochemical processes but can also affect the health of plants through interactions (Oliveira et al. 2009). However, microbial activity can be distorted by various types of contaminants dissipating into the terrestrial environment, such as fungicides and other substances. Changes in microbial activity can be manifested by their lower count and biodiversity, which has a strong impact on maintaining correct soil quality (Saha et al. 2012). According to Kucharski and Wyszkowska (2008), pesticides can have varying effects on the growth and development of microorganisms, and their impact is not only determined by the type and application rate but also depends on microorganisms dwelling in the soil ecosystem. Findings from studies show that azoxystrobin changes the counts of organotrophic bacteria, actinomycetes and fungi (Table 4). Microbial counts significantly correlated with the dose of azoxystrobin (the percentage of variance ranged from 8.4 to 45.7 %) and soil incubation time (the percentage of variance ranged from 10.9 to 61.2 %). A significant decrease in the counts of fungi was observed after treatment of soil with azoxystrobin. The strongest effect was observed for the highest dose (22.50 mg kg^−1^), which reduced the fungi counts on day 30 by 0.294 log, on day 60 by 0.397 log and on day 90 by 0.426 log. A study by Crouzet et al. (2010) revealed decreased counts of fungi in soil treated with mesotrione (herbicide). Walia et al. (2014) reported that mancozeb (fungicide) applied to soil in doses of between 10 and 2000 mg kg^−1^ had a strong inhibitory effect on fungi. Similar findings, but after the treatment of soil with herbicides, were made by Baćmaga et al. (2015), who tested a mixture of diflufenican + mesosulfuron-methyl + iodosulfuron-methyl-sodium, and Araújo et al. (2003), who tested the effects of glyphosate. On the other hand, Martinez et al. (2008) found no significant differences in fungal counts in soil contaminated with sulfentrazone. The effects of azoxystrobin on the counts of oligotrophic bacteria and actinomycetes were variable. On day 30 of the experiment, a stimulating effect of azoxystrobin on these microorganisms was found. The highest count of organotrophic bacteria was recorded for dose 22.50 mg kg^−1^ (increase by 0.200 log) and of actinomycetes for dose 0.075 mg kg^−1^ (increase by 0.215 log). On days 60 and 90, azoxystrobin inhibited the growth of organotrophic bacteria and actinomycetes, as indicated by negative values of correlation coefficients. The microbial counts decreased in proportion to the dose of the applied fungicide. Reduction in the counts of bacteria and actinomycetes after treatment with herbicides were also found by Baćmaga et al. (2014b) in soil contaminated with metazachlor and by Baćmaga et al. (2015) after soil treatment with a mixture of diflufenican + mesosulfuron-methyl + iodosulfuron-methyl-sodium. Similar findings were made by Sebiomo et al. (2011) and Kucharski and Wyszkowska (2008). The study by Sebiomo et al. (2011) analysed the influence of atrazine, primextar, paraquat and glyphosate on soil microorganisms, while Kucharski and Wyszkowska (2008) investigated the effects of Apyros 75 WG containing sulfosulfuron as an active substance. Walia et al. (2014) observed a harmful effect of mancozeb (fungicide) on the populations of actinomycetes caused by all doses (10 to 2000 mg kg^−1^), while bacteria were susceptible to treatment with fungicide at the highest doses (1000 and 2000 mg kg^−1^). Increases in actinomycetes counts in response to increasing doses of herbicides were reported by Araújo et al. (2003) in a study with glyphosate, and Tomkiel et al. (2014), who treated soil with a mixture of pethoxamid and terbuthylazine (Successor T 550 SE). These different effects of pesticides on soil microorganisms may result from the different chemical structures of the active substance or the tolerability of microorganisms to these products. Pesticides, for some microorganisms, may be a suitable nutrient or source of energy, but may be toxic to others and cause disorders in cell metabolism (Crouzet et al. 2010; Singh and Goshal 2010). The response of the tested microorganisms to soil contamination with azoxystrobin was confirmed by Ward’s cluster analysis (Fig. 1). Two clusters were identified on the dendrogram. The first cluster comprised organotrophic bacteria and actinomycetes, and the second comprised fungi. It can be concluded from the dendrogram that organotrophic bacteria and actinomycetes responded in a similar manner to the treatment of soil with azoxystrobin.

**Table 4:** Microorganisms counts in soil contaminated with azoxystrobin, log CFU kg^−1^ soil DM

Dose of azoxystrobin (mg kg^−1^)	Organotrophic bacteria			Actinomycetes			Fungi		
	Soil incubation time, days								
	30	60	90	30	60	90	30	60	90
0.000	10.186^b^	10.474^a^	10.459^a^	9.938^c^	10.288^a^	10.293^a^	7.153^abc^	7.348^ab^	7.440^a^
0.075	10.294^ab^	10.420^ab^	10.425^ab^	10.153^ab^	10.199^ab^	10.232^ab^	7.112^abc^	7.26^abc^	7.285^abc^
2.250	10.315^ab^	10.311^ab^	10.428^ab^	10.087^bcd^	10.190^ab^	10.217^ab^	6.878^bc^	7.128^abc^	7.203^abc^
11.25	10.384^ab^	10.270^ab^	10.395^ab^	9.992^cd^	10.168^cd^	10.206^ab^	6.894^bc^	7.073^abc^	7.112^abc^
22.50	10.406^ab^	10.192^b^	10.282^ab^	9.941^c^	10.137^abc^	10.175^ab^	6.858^bc^	6.951^bc^	7.014^abc^
$\overline{x}$	10.320	10.340	10.400	10.020	10.200	10.230	7.000	7.170	7.220
*r*	0.813	−0.891*	−0.963*	−0.547	−0.762	−0.791	−0.709	−0.902*	−0.876*

Homogeneous groups are denoted with the same letters within microbial groups for all dates of analysis, in columns

$\overline{x}$ mean, *r* coefficient of correlation at *p = 0.01

*p = 0.01

**Fig. 2:**
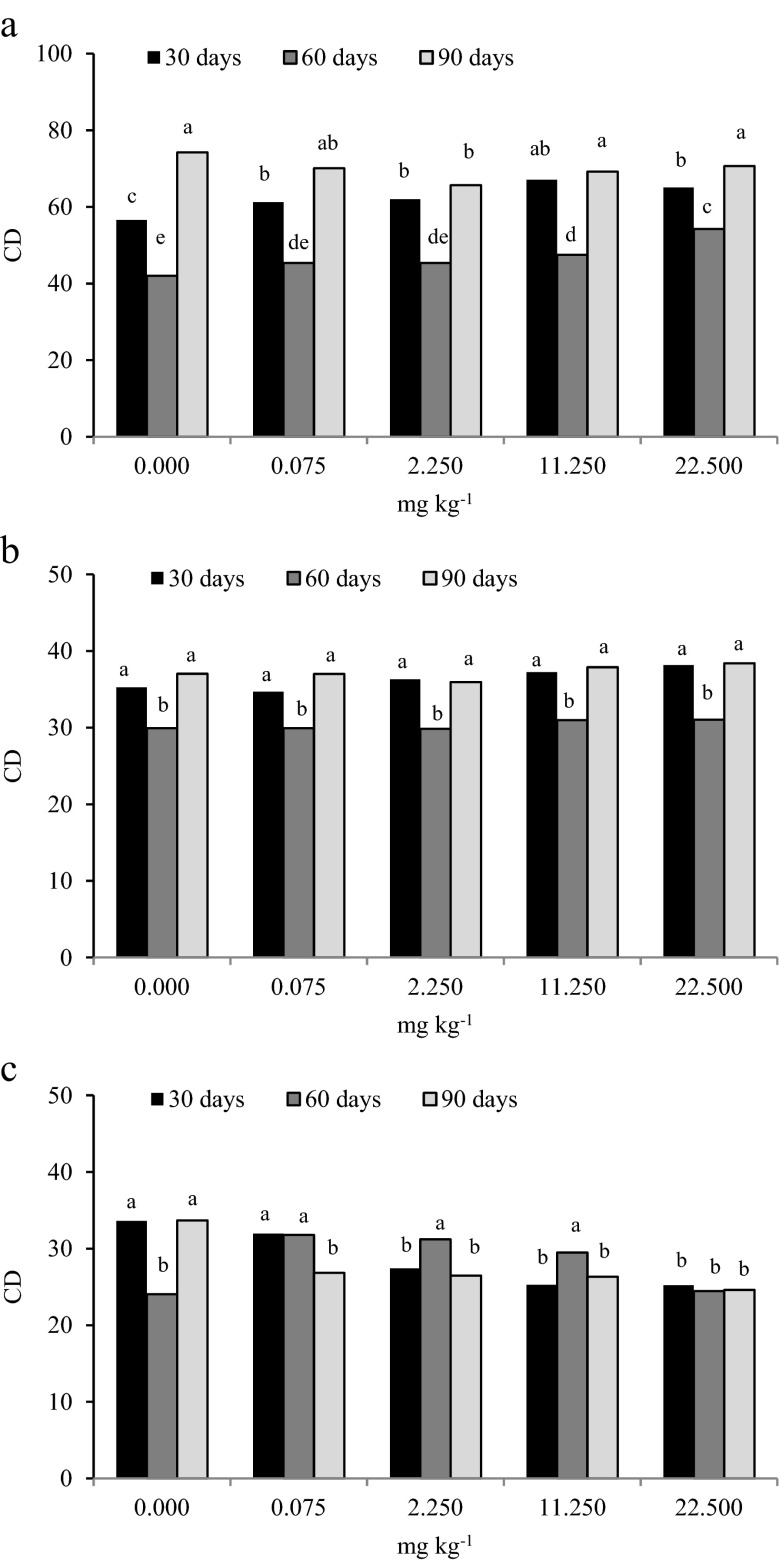
The effect of azoxystrobin on development index (CD) of **a** organotrophic bacteria, **b** actinomycetes, **c** fungi. Explanation: Homogeneous groups are denoted with the same letters within microbial groups for all dates of analysis

**Fig. 3:**
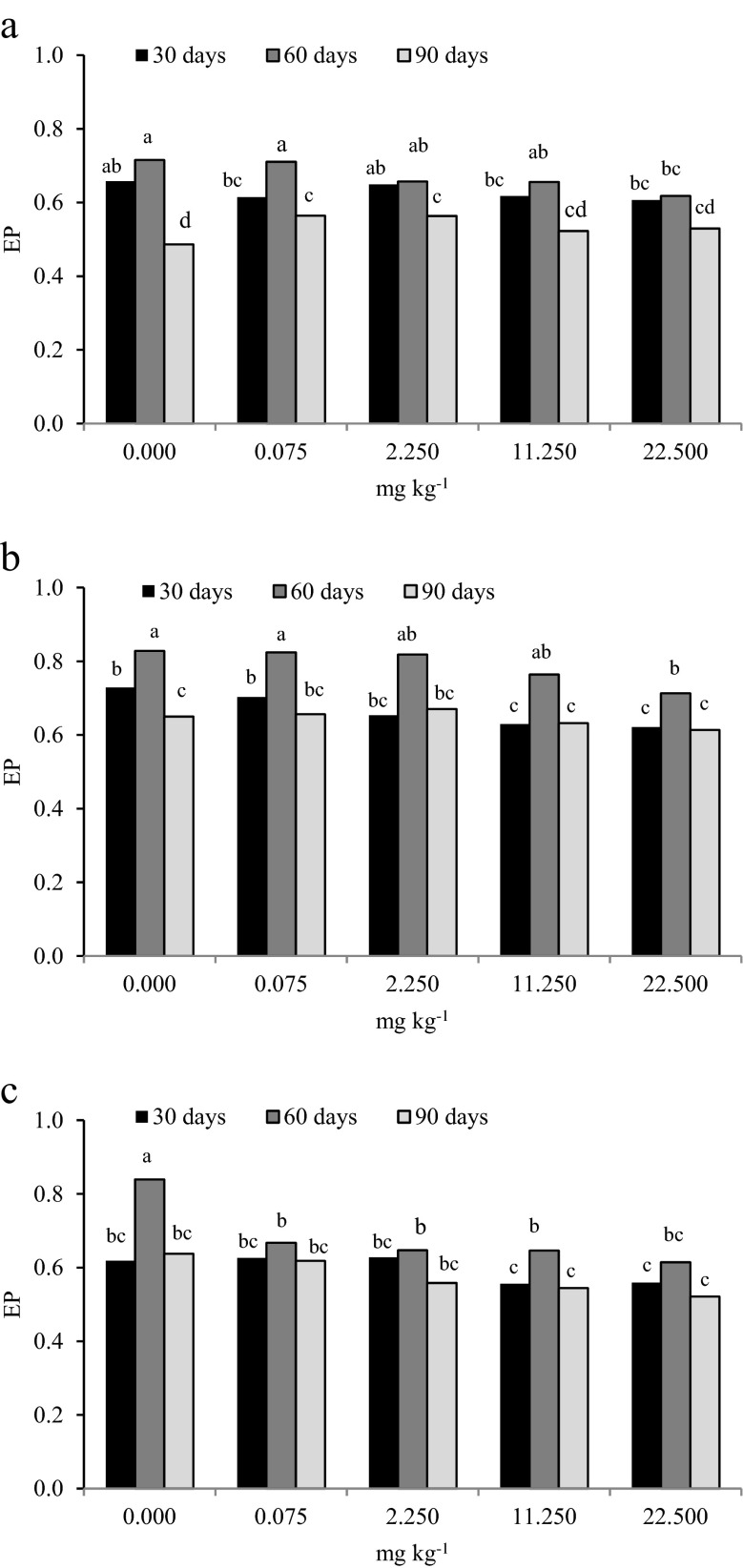
The effect of azoxystrobin on ecophysiological index (EP) of **a** organotrophic bacteria, **b** actinomycetes, **c** fungi. Explanation: Homogeneous groups are denoted with the same letters within microbial groups for all dates of analysis

Pesticides affect soil microbial counts but can also lead to changes in microbial biodiversity (Demenaou et al. 2004; Ratcliff et al. 2006). In our study, the microbial biodiversity was determined based on the colony development (CD) index and the ecophysiological (EP) index. CD values indicated that azoxystrobin had a significant effect on the biodiversity of the tested soil microorganisms (Fig. 2). The highest mean values of the CD index were found for organotrophic bacteria (59.762) and the lowest for fungi (28.160). With respect to the soil incubation time, the colony development index for organotrophic bacteria and actinomycetes was the highest on day 90 (mean 69.979 for organotrophic bacteria and 37.259 for actinomycetes). The CD index for moulds was the highest on day 30 of the experiment (mean 28.695). Treatment of soil with azoxystrobin at a dose of 22.50 mg kg^−1^ increased CD values for organotrophic bacteria on days 30 and 60 (by 14.98 % on day 30, and by 29.11 % on day 60). The CD index for actinomycetes increased on all test dates, particularly on day 30 (by 8.31 %). Fungi were found to be very susceptible to high doses of azoxystrobin. A significant decrease in the CD index vs. control soil samples was observed on days 30 and 90 following the application of fungicide at a dose of 22.50 mg kg^−1^ (decrease by 25.0 and 26.97 %, respectively). Azoxystrobin also caused changes in the EP index for microorganisms (Fig. 3). The lowest EP index for microorganisms was found on day 90 (0.533 for organotrophic bacteria, 0.644 for actinomycetes and 0.576 for moulds). Azoxystrobin at the highest dose (22.50 mg kg^−1^) caused a significant decrease in the EP index on all test dates. The only exception was organotrophic bacteria on day 90, for which an increase in the EP index by 8.16 % vs. control was found. Generally, soil microorganisms respond promptly to environmental changes and are therefore considered robust indicators of soil quality and fertility (Serrano et al. 2009). Modifications in microbial composition manifested by changes in the proportion of r-strategists to K-strategists are caused by pesticides dissipating to the soil and are often used in tests evaluating the impact of pesticides on microbial soil parameters. Sarathchandra et al. (1997) reported that an increase in the CD index indicates the predominance of fast-growing microbes (r-strategists) over slowly-growing ones (K-strategists). On the other hand, a decrease in the EP index may indicate the elimination of microbial species susceptible to stressors, including pesticides, by more resistant ones (De Leij et al. 1993). In our study, the values of the CD and EP indices in contaminated soil were generally lower in comparison to the control sample. These values differed depending on the dose of azoxystrobin, soil incubation time and the group of analysed soil microorganisms. Similar findings were made in a study by Ros et al. (2006) investigating the effects of atrazine.

### Soil enzymes

Enzymatic soil activity is one of the key parameters used in assessing the quality and fertility of soil contaminated with pesticides and other chemicals (Baćmaga et al. 2012; Kucharski et al. 2009; Singh and Kumar 2008; Wyszkowska 2002; Zhang et al. 2014). Pesticides can also have a direct impact on enzymes by inhibiting their catalytic potential, or by the involvement in the modification of microbial activity (Floch et al. 2011). Azoxystrobin inhibits the enzymatic activity of soil, as indicated by negative correlation coefficients between doses and the activity levels of these enzymes (Table 5). Fungicide dose determined from 0.6 % (alkaline phosphatase) to 82.4 % (acid phosphatase) of changes in enzymatic activity, and soil incubation time determined from 3.7 % (catalase) to 99.0 % (alkaline phosphatase) of changes. The most significant changes in enzymatic activity were caused by the highest dose (22.50 mg kg^−1^), which significantly reduced the mean activity of dehydrogenases (by 7.04 %), catalase (by 22.86 %), urease (by 31.25 %), acid phosphatase (by 20.45 %) and alkaline phosphatase (by 11.78 %). Literature data suggest that dehydrogenases (Baćmaga et al. 2012; Baćmaga et al. 2014b; Baćmaga et al. 2015; Demenaou et al. 2004; Kucharski and Wyszkowska 2008; Monkiedje et al. 2002), β-glucosidase (Demenaou et al. 2004; Monkiedje et al. 2002) and alkaline phosphatase (Monkiedje et al. 2002) are the most susceptible to pesticides. Moreover, Chen et al. (2001) demonstrated that benomyl and captan (fungicides) had the strongest inhibitory effect on the activity of dehydrogenases and acid phosphatase. In our study, azoxystrobin had the weakest effect on the activity of dehydrogenases, and the strongest negative effect on urease in the treated soil. Studies by Tomkiel et al. (2014) showed that a mixture of two herbicides, terbuthylazine and pethoxamid, also inhibited the activity of urease. A similar response of urease was observed by Sukul (2006) after treating soil with metalaxyl (fungicide). Jastrzębska and Kucharski (2007), in an experiment with fungicides (cyprodinil and a mixture of dimoxystrobin + epoxiconazole), noted the negative effects of these pesticides on the activity of dehydrogenases and urease. In our study, the activity of catalase, acid phosphatase and alkaline phosphatase also decreased under the influence of high doses of azoxystrobin, but the effect was less pronounced than that observed for urease. Baćmaga et al. (2014b) found metazachlor (herbicide) to be a potent inhibitor of catalase, acid phosphatase, alkaline phosphatase, arylsulfatase and β-glucosidase. Wyszkowska and Kucharski (2004) investigated the effects of Triflurotox 250 EC (herbicide) in application doses from 1.5 to 12 mm^3^ kg^−1^ and reported its negative impact not only on the activity of dehydrogenases and urease but also on acid and alkaline phosphatases. Yao et al. (2006) demonstrated that acetamipiryd (insecticide) did not have a significant impact on the activity of catalase and urease but inhibited the activity of phosphatases. Walia et al. (2014) treated soil with mancozeb in doses from 10 to 2000 mg kg^−1^ and observed the destructive activity of this fungicide on the activity of phosphatases, amylase and invertase. Baćmaga et al. (2012) tested the effects of carfentrazone-ethyl (herbicide) on dehydrogenases, acid phosphatase and alkaline phosphatase and found no changes in their activity levels. Regardless of the dose, the lowest activity of catalase, urease, acid phosphatase and alkaline phosphatase was observed on day 90 and of dehydrogenases on day 30.

**Table 5:** Enzymatic activity in soil contaminated with azoxystrobin, 1 kg DM h^−1^

Dose of azoxystrobin (mg kg^−1^)	Dehydrogenases			Catalase			Urease			Acid phosphatase			Alkaline phosphatase		
	μMol TPF			Mol 0_2_			mMol N-NH_4_			mMol PNP					
	Soil incubation time, days														
	30	60	90	30	60	90	30	60	90	30	60	90	30	60	90
0.000	5.310^e^	9.151^ab^	7.255^c^	0.360^a^	0.346^ab^	0.334^abc^	0.175^a^	0.165^ab^	0.136^cde^	0.910^a^	0.878^ab^	0.863^abc^	1.276^c^	2.259^a^	0.470^ef^
0.075	5.412^e^	9.529^a^	6.645^d^	0.335^abc^	0.331^abc^	0.331^abc^	0.164^ab^	0.165^ab^	0.132^de^	0.902^ab^	0.837^abcd^	0.828^bcd^	1.091^d^	2.282^a^	0.496^e^
2.250	5.386^e^	9.676^a^	6.621^d^	0.329^abcd^	0.302^bc^	0.321^abc^	0.148^cd^	0.134^de^	0.129^de^	0.869^abc^	0.790^cde^	0.774^def^	1.064^d^	2.229^a^	0.490^e^
11.25	5.211^e^	9.525^a^	6.489^d^	0.299^bc^	0.290^bcd^	0.272^cd^	0.137^cde^	0.130^de^	0.109^ef^	0.760^defg^	0.740^efg^	0.716^efg^	1.055^d^	2.130^b^	0.431^ef^
22.50	5.087^e^	8.808^b^	6.309^d^	0.275^bcd^	0.272^cd^	0.262^d^	0.131^de^	0.110^ef^	0.092^f^	0.705^fg^	0.694^fg^	0.686^g^	1.043^d^	2.090^b^	0.406^g^
$\overline{x}$	5.281	9.338	6.664	0.320	0.308	0.304	0.151	0.141	0.119	0.829	0.788	0.773	1.106	2.198	0.458
*r*	−0.945*	−0.652	−0.727	−0.946*	−0.884*	−0.952*	−0.860	−0.892*	−0.993*	−0.978*	−0.925*	−0.908*	−0.555	−0.961*	−0.942*

Homogeneous groups are denoted with the same letters within soil enzymes for all dates of analysis, in columns

$\overline{x}$ mean, *r* coefficient of correlation at *p = 0.01

*p = 0.01

**Fig. 4:**
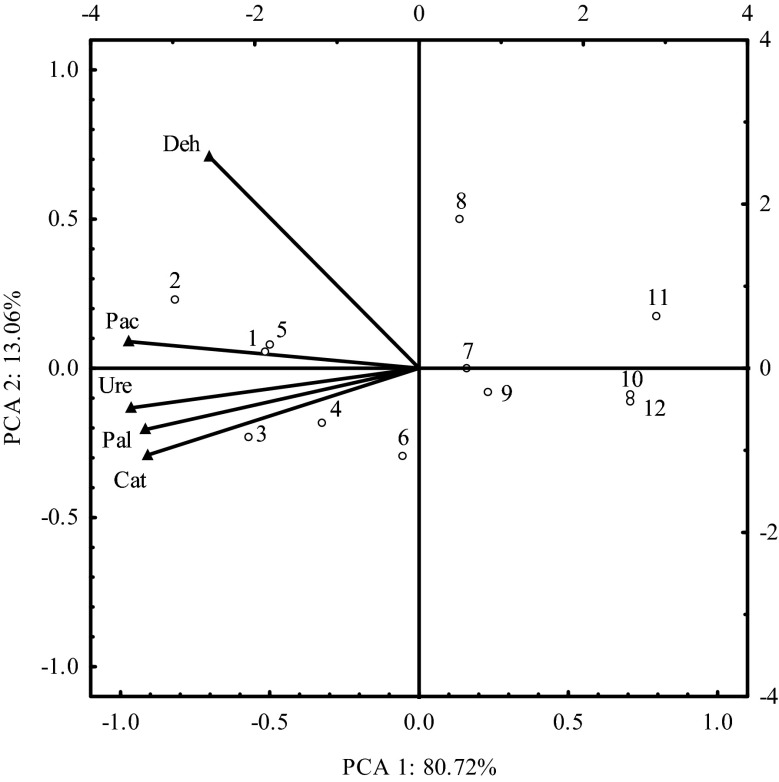
Resistance of soil enzymes to contamination with azoxystrobin, determined by PCA. Explanation: vectors represent the primary variable: *Deh* dehydrogenases, *Cat* catalase, *Ure* urease, *Pac* acid phosphatase, *Pal* alkaline phosphatase; cases: 1–3 (0.075 mg kg^−1^); 4–6 (2.250 mg kg^−1^); 7–9 (11.25 mg kg^−1^); 10–12 (22.50 mg kg^−1^)

Determination of the soil resistance index and soil resilience index helps to identify whether or not a particular ecosystem exposed to various stressors can remain stable and maintain appropriate balance (Griffiths and Philippot 2013; Orwin and Wardle 2004; Orwin and Wardle 2005; Orwin et al. 2006). In our study, the resistance of soil enzymes depended both on the dose of azoxystrobin and soil incubation time (Table 6). Treatment of soil with higher doses of the tested fungicide reduced RS values for all the analysed enzymes. However, urease was found most susceptible to the fungicide, particularly on day 60, when the RS value decreased from 0.908 to 0.337. In contrast to that, dehydrogenases showed the highest resistance to increased doses of azoxystrobin. Treatment of soil with a dose of 2.25 mg kg^−1^ decreased the RS value on day 60 from 0.925 to 0.898. In terms of resistance to the tested fungicide, enzymes were categorized in the following order: dehydrogenases (0.874) > alkaline phosphatase (0.810) > acid phosphatase (0.746) > catalase (0.718) > urease (0.646). Tomkiel et al. (2014) also noticed that urease was the most susceptible to the effects of a mixture containing two herbicides—pethoxamid and terbuthylazine. Baćmaga et al. (2015) analysed the effects of soil treatment with a mixture of different herbicides (diflufenican + mesosulfuron-methyl + iodosulfuron methyl-sodium) on the enzymatic activity of soil and found the greatest resistance for urease and the lowest resistance for dehydrogenases. In another study, Be’caerta et al. (2006) tested 2,4-D and found that β-glucosidase and arylsulfatase were most susceptible to soil contamination with this herbicide. Baćmaga et al. (2014a), in an experiment with three herbicides (Fuego 500 SC, Alister Grande 190 OD and Lumax 537.5 SE), observed that β-glucosidase, followed by arylsulfatase, were the most resistant to Alister Grande 190 OD. The resistance (RS) of soil enzymes to contamination with azoxystrobin revealed by PCA is presented in Fig. 4. The first two principal components accounted for 93.78 % of the total variance. Around the first principal component, a homogeneous cluster was formed, including catalase, urease, acid phosphatase and alkaline phosphatase. There was a negative correlation between this variable and the resistance of the enzymes and a positive correlation between the resistance of enzymes. Dehydrogenases were positively correlated with the second principal component and negatively with the first component. The position of vectors in four quadrants of the coordinate system indicates different effects of azoxystrobin on the resistance of soil enzymes. As shown in the figure, the resistance of all the analysed enzymes depended on the level of soil contamination with this fungicide. The lowest resistance was found for enzymes in soil treated with azoxystrobin at doses of 11.25 mg kg^−1^ and 22.50 mg kg^−1^. Azoxystrobin contributed to long-term changes in the analysed soil (Table 7). In some samples, the soil resilience (RL) index was negative, reflecting the progressingly harmful effects of fungicide on soil biology during the experimental period. RL values ranged between −0.866 and 0.374. Mean values of this index indicated that the balance was recovered in the longest time by catalase (RL = −0.689) and in the shortest time by alkaline phosphatase (RL = 0.296). The positive values of the RL index demonstrated the ability of soil to mitigate the stressful effects of pesticides. Baćmaga et al. (2014a), in an experiment with three herbicides (Fuego 500 SC, Alister Grande 190 OD and Lumax 537.5 SE), observed that longer time was required for arylsulfatase to recover balance than for β-glucosidase. However, the strongest negative effect on the value of the RL index was produced by Fuego 500 SC, containing metazachlor. Tomkiel et al. (2014) studied the effects of Successor T 550 SE (herbicide) and demonstrated the shortest recovery time for urease and the longest for arylsulfatase.

**Table 6:** Resistance (RS) enzymes to soil contaminated with azoxystrobin

Dose of azoxystrobin (mg kg^−1^)	Dehydrogenases			Catalase			Urease			Acid phosphatase			Alkaline phosphatase		
	Soil incubation time, days														
	30	60	90	30	60	90	30	60	90	30	60	90	30	60	90
0.075	0.949^a^	0.925^ab^	0.817^c^	0.863^ab^	0.870^ab^	0.878^ab^	0.877^ab^	0.908^a^	0.803^b^	0.913^a^	0.893^ab^	0.901^a^	0.711^cd^	0.964^a^	0.905^ab^
2.250	0.884^ab^	0.898^ab^	0.828^ab^	0.832^b^	0.610^b^	0.917^a^	0.690^c^	0.635^c^	0.895^a^	0.893^ab^	0.802^b^	0.799^b^	0.667^cd^	0.968^a^	0.922^ab^
11.25	0.931^ab^	0.920^ab^	0.793^c^	0.667^c^	0.682^c^	0.626^c^	0.573^c^	0.567^c^	0.603^c^	0.675^c^	0.690^c^	0.663^c^	0.654^d^	0.886^ab^	0.836^b^
22.50	0.878^ab^	0.918^ab^	0.743^c^	0.529^d^	0.573^d^	0.568^d^	0.501^c^	0.337^d^	0.366^d^	0.552^d^	0.584^d^	0.592^d^	0.633^d^	0.851^b^	0.727^c^
$\overline{x}$	0.911	0.915	0.795	0.723	0.684	0.747	0.660	0.612	0.667	0.758	0.742	0.739	0.666	0.917	0.847
*r*	−0.519	0.210	−0.972*	−0.997*	−0.656	−0.933*	−0.933*	−0.918*	−0.969*	−0.986*	−0.975*	−0.951*	−0.874	−0.972*	−0.984*

Homogeneous groups are denoted with the same letters within soil enzymes for all dates of analysis, in columns

$\overline{x}$ mean, *r* coefficient of correlation at *p = 0.01

*p = 0.01

**Table 7:** Resilience index (RL) of soil contaminated with azoxystrobin on day 90 of the experiment

Dose of azoxystrobin (mg kg^−1^)	Dehydrogenases	Catalase	Urease	Acid phosphatase	Alkaline phosphatase
0.075	−0.467	−0.818	−0.491	−0.786	0.235
2.250	−0.467	−0.866	−0.468	−0.647	0.243
11.25	−0.508	−0.624	−0.225	−0.536	0.330
22.50	−0.551	−0.449	−0.062	−0.485	0.374
$\overline{x}$	−0.498	−0.689	−0.312	−0.614	0.296
*r*	−0.996*	0.979*	0.991*	0.902	0.982*

$\overline{x}$ mean, *r* coefficient of correlation at *p = 0.01

*p = 0.01

### Identification of DNA from bacteria and fungi

Microorganisms play a key role in the biodegradation of pesticides and other chemicals in the soil ecosystem. Therefore, the use of pesticides requires an understanding of their effects on soil-dwelling microorganisms and the potential consequences of their inappropriate application. The application of high doses of pesticides in microbiological experiments allows for the identification of microbial strains with a high degradation potential and those useful for the bioremediation of soils contaminated with these substances (Chen et al. 2011; Jezierska-Tys and Rutkowska 2013). In this study, four bacterial strains of *Bacillus* spp. and two fungal strains of *Aphanoascus* spp. (Table 8) potentially able to degrade azoxystrobin used at a dose of 22.50 mg kg^−1^ DM were isolated from soil. Azoxystrobin is one of the most important strobilurin-derived fungicides used on a large scale to protect plants against fungal pathogens. The isolated bacterial strains were identified as *Bacillus cereus* (KC848897.1), *Bacillus weihenstephanensis* (KF831381.1), *Bacillus* sp. (LM655314.1) and *Bacillus megaterium* (KJ843149.1). Cycoń et al. (2011) analysed soil contaminated with methyl thiophanate and isolated two bacterial strains, TDS and TDS-1, classified to the genera *Enterobacter* and *Bacillus.* In another study, Chennappa et al. (2014) concluded that *Azotobacter* spp. bacteria are able to degrade pesticides. The researchers demonstrated that 13 out of 14 isolated strains were able to grow on culture media containing herbicides (pendimethalin and glyphosate) and insecticides (chlorpyrifos and phorate). Ahemad and Khan (2011) tested tebuconazole (fungicide) in doses of 100, 200 and 300 mg kg^−1^ and isolated *Rhizobium* strain MRP1. They also observed that the treatment of soil contaminated with tebuconazole increased pea production. Studies by Zhang et al. (2012) demonstrated that certain microbial species, such as *Rhodobacter sphaeroides* W16 and *Acinetobacter lwoffii* DNS32 isolates, may be useful in the bioremediation of soils contaminated with atrazine (herbicide). Researchers also concluded that atrazine produced a greater inhibition of growth in *Bacillus subtilis* B19. To date, there have been no available reports on azoxystrobin-degrading fungal strains. Therefore, our study was aimed at isolating fungi able to degrade this fungicide. Based on DNA sequencing of the ITS region, two strains, *Aphanoascus terreus* (AB861677.1) and *Aphanoascus fulvescens* (JN943451.1), were isolated from sandy clay loam contaminated with azoxystrobin at a dose of 22.500 mg kg^−1^.

**Table 8:** Identification of bacteria (based on the 16S rRNA gene sequence) and fungi (based on the ITS region sequence) isolated from soil

Dose of azoxystrobin	Strain number	Homology to		
mg kg^−1^		Species	Strain	%
Bacteria				
0.00	B1	*Bacillus anthracis*	KF475884.1	99
	B2	*Bacillus cereus*	KF010349.1	99
	B3	*Bacillus* sp.	JN872500.1	100
	B4	*Bacillaceae bacterium*	DQ490406.1	99
0.075	B5	*Bacillus* sp.	KM083527.1	100
	B6	*Bacillus* sp.	KF956561.1	99
	B7	*Bacillus megaterium*	KJ919967.1	100
	B8	*Bacillus simplex*	GU969132.1	99
22.50	B9	*Bacillus cereus*	KC848897.1	99
	B10	*Bacillus weihenstephanensis*	KF831381.1	99
	B11	*Bacillus* sp.	LM655314.1	100
	B12	*Bacillus megaterium*	KJ843149.1	100
Fungi				
0.00	G1	*Acremonium cellulolyticus*	KF811039.1	100
	G2	*Talaromyces pinophilus*	KF751644.1	100
	G3	*Penicillium* sp.	GU973745.1	99
	G4	*Trichoderma viride*	GU048860.1	99
0.075	G5	*Penicillium chrysogenum*	KF039676.1	99
	G6	*Penicillium* sp.	AB734793.1	99
	G7	*Acremonium cellulolyticus*	GU479898.1	99
22.50	G8	*Aphanoascus terreus*	AB861677.1	99
	G9	*Aphanoascus fulvescens*	JN943451.1	99

**Fig. 5:**
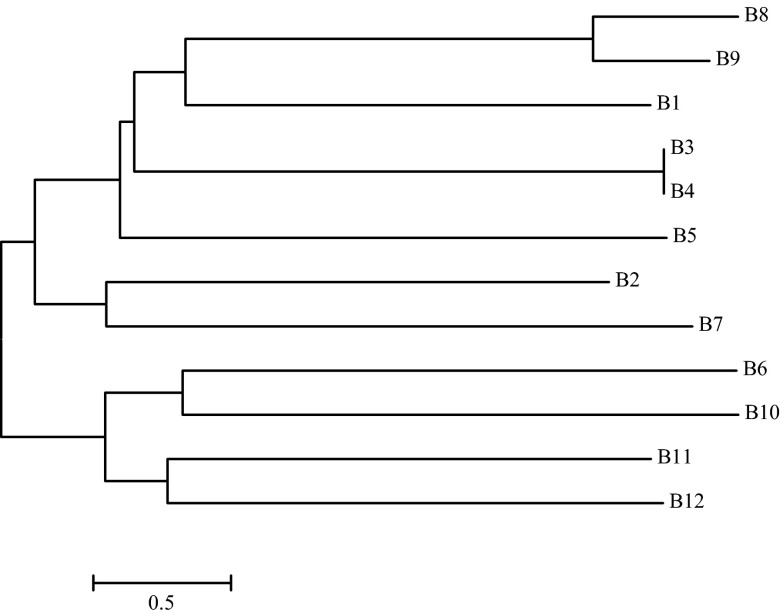
A phylogenetic tree of bacterial strains isolated from soil, prepared based on the comparison of the 16S rRNA gene sequence. Explanation: B1, *Bacillus anthracis*; B2, *Bacillus cereus*; B3, *Bacillus* sp.; B4, *Bacillaceae bacterium*; B5, *Bacillus* sp.; B6, *Bacillus* sp.; B7, *Bacillus megaterium*; B8 *Bacillus simplex*; B9, *Bacillus cereus*; B10, *Bacillus weihenstephanensis*; B11, *Bacillus* sp.; B12, *Bacillus megaterium*

A phylogenetic tree representing the linkage distance between the analysed bacterial strains (Fig. 5) prepared based on the analysis of the 16S rRNA gene sequence indicated three groups with a high level of similarity. The first group comprised two more or less strongly related sub-groups, i.e. B11 and B12 strains in the first sub-group, and B6 and B10 strains in the second one. The genetic distance between these strains was 1.8 and 2.0 %, respectively. The second group comprised B2 and B7 strains (genetic distance 2.1 %). The third group comprised four sub-groups: B5 strain in the first, B3 and B4 in the second, B1 in the third, and B8 and B9 in the fourth.

**Fig. 6:**
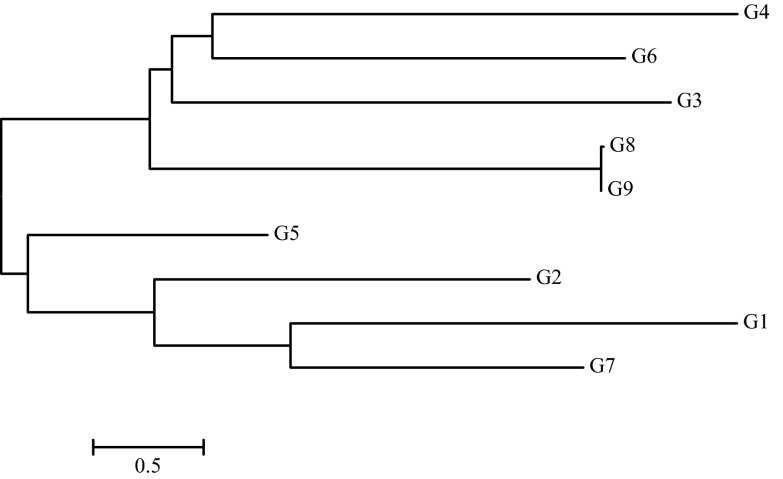
A phylogenetic tree of fungal strains isolated from soil, prepared based on the comparison of the ITS region sequence. Explanation: G1, *Acremonium cellulolyticus*; G2, *Talaromyces pinophilus*; G3, *Penicillium* sp.; G4, *Trichoderma viride*; G5, *Penicillium chrysogenum*; G6, *Penicillium* sp.; G7, *Acremonium cellulolyticus*; G8, *Aphanoascus terreus*; G9, *Aphanoascus fulvescens*

The phylogenetic tree for fungi obtained based on the analysis of nucleotide sequences from the ITS region is presented in Fig. 6. Fungi isolated from soil were classified to the genera *Trichoderma*, *Penicillium*, *Aphanoascus*, *Talaromyces* and *Acremonium*. The G1 isolate had 100 % and G7 99 % similarity with the nucleotide sequence of *Acremonium cellulolyticus.* Other isolated strains had very high levels of similarity (99 %) with other species: G2 with *Talaromyces pinophilus*, G3 and G6 with *Penicillium* sp., G4 with *Trichoderma viride*, G5 with *Penicillium chrysogenum*, G8 with *Aphanoascus terreus* and G9 with *Aphanoascus fulvescens*. A dendrogram indicated two major groups of genetically related fungi, i.e. G1, G2, G5 and G7 strains in the first group, and G3, G4, G6, G8 and G9 strains in the second group.

## Conclusions

Azoxystrobin has a harmful effect on soil microorganisms and their biodiversity, as well as enzymatic activity and resistance of soil. The microbial and biochemical soil indices identified in the study provided necessary information about soil quality and fertility. The calculated predicted environmental concentration (PEC) of azoxystrobin in soil confirms the fact that the use of this fungicide in contaminating doses creates a risk to living organisms. These findings suggest that azoxystrobin designed for the control of fungal diseases in crops and vegetables should be used carefully and according to the manufacturer’s recommendations. Its use in increased doses distorts the homeostasis of soil determined based on the activity of soil microorganisms, which can have a strong impact on plant growth and yield. Bacterial and fungal strains isolated from soil show the adaptability to contamination of soil with azoxystrobin but also with other strobilurin-derived substances. Because of their degrading potential, these microorganisms can be considered when developing strategies for the bioremediation of soils contaminated with pesticides.
